# Conditional Disabled-1 Deletion in Mice Alters Hippocampal Neurogenesis and Reduces Seizure Threshold

**DOI:** 10.3389/fnins.2016.00063

**Published:** 2016-02-25

**Authors:** Matthew J. Korn, Quinton J. Mandle, Jack M. Parent

**Affiliations:** ^1^Department of Neurology, University of Michigan Medical CenterAnn Arbor, MI, USA; ^2^VA Ann Arbor Healthcare SystemAnn Arbor, MI, USA

**Keywords:** neural stem cells, adult neurogenesis, temporal lobe epilepsy, pilocarpine

## Abstract

Many animal models of temporal lobe epilepsy (TLE) exhibit altered neurogenesis arising from progenitors within the dentate gyrus subgranular zone (SGZ). Aberrant integration of new neurons into the existing circuit is thought to contribute to epileptogenesis. In particular, adult-born neurons that exhibit ectopic migration and hilar basal dendrites (HBDs) are suggested to be pro-epileptogenic. Loss of reelin signaling may contribute to these morphological changes in patients with epilepsy. We previously demonstrated that conditional deletion of the reelin adaptor protein, disabled-1 (Dab1), from postnatal mouse SGZ progenitors generated dentate granule cells (DGCs) with abnormal dendritic development and ectopic placement. To determine whether the early postnatal loss of reelin signaling is epileptogenic, we conditionally deleted Dab1 in neural progenitors and their progeny on postnatal days 7–8 and performed chronic video-EEG recordings 8–10 weeks later. Dab1-deficient mice did not have spontaneous seizures but exhibited interictal epileptiform abnormalities and a significantly reduced latency to pilocarpine-induced status epilepticus. After chemoconvulsant treatment, over 90% of mice deficient for Dab1 developed generalized motor convulsions with tonic-clonic movements, rearing, and falling compared to <20% of wild-type mice. Recombination efficiency, measured by Cre reporter expression, inversely correlated with time to the first sustained seizure. These pro-epileptogenic changes were associated with decreased neurogenesis and increased numbers of hilar ectopic DGCs. Interestingly, neurons co-expressing the Cre reporter comprised a fraction of these hilar ectopic DGCs cells, suggesting a non-cell autonomous effect for the loss of reelin signaling. We also noted a dispersion of the CA1 pyramidal layer, likely due to hypomorphic effects of the conditional Dab1 allele, but this abnormality did not correlate with seizure susceptibility. These findings suggest that the misplacement or reduction of postnatally-generated DGCs contributes to aberrant circuit development and hyperexcitability, but aberrant neurogenesis after conditional Dab1 deletion alone is not sufficient to produce spontaneous seizures.

## Introduction

Mesial temporal lobe epilepsy (mTLE) is a common and often intractable focal epilepsy (Bender et al., 2004; Dube et al., 2012). Structural changes in the hippocampal dentate gyrus (DG) are implicated in mTLE pathogenesis, though the mechanisms underlying these changes are poorly understood. Neurogenesis is markedly disrupted in rodent models of mTLE (Parent et al., 1997, 2006; Jessberger et al., 2005, 2007; Shapiro et al., 2005; Murphy et al., 2011, 2012), leading to the idea that aberrantly integrated adult-born neurons (namely dentate granule cells, DGCs) produce seizures (Parent and Kron, 2012; Pun et al., 2012; Bielefeld et al., 2014; Cho et al., 2015) and contribute to associated comorbidities such as cognitive dysfunction and depression (Groticke et al., 2007; Muller et al., 2009; Zhang et al., 2010; Lesting et al., 2011; Levin et al., 2012; Klein et al., 2015).

The role of adult DGC neurogenesis is complex and there are several competing hypotheses as to how continuous integration of new neurons might be implicated in seizure development. Intact adult neurogenesis may mitigate acute seizure susceptibility (Iyengar et al., 2015) and suppressing adult neurogenesis with brain irradiation can exacerbate kindling progression (Raedt et al., 2007; Pekcec et al., 2011). However, the development of epilepsy after an acute insult, such as status epilepticus (SE), is attenuated if the seizure-induced increase in adult neurogenesis is inhibited by antimitotic agents (Jung et al., 2006; Sugaya et al., 2010) or pharmacogenetic methods (Cho et al., 2015). Perhaps most compelling is the finding that disrupting the integration of a subset of postnatal-born DGCs by suppressing phosphatase and tensin homolog (PTEN) signaling is sufficient to produce spontaneous seizures in mice, and the affected DGCs exhibit many of the aberrant changes seen in animal models of mTLE (Pun et al., 2012). Thus, there is an interest in identifying signaling pathways that regulate DGC neurogenesis and whose disruption might produce mTLE.

Reelin-expressing interneurons persist throughout the adult DG (Alcantara et al., 1998; Pesold et al., 1998; Gong et al., 2007) and reelin expression is decreased in human and experimental mTLE (Gong et al., 2007; Haas and Frotscher, 2010). It is proposed that altered neurogenesis in mTLE may arise in part due to disrupted reelin signaling (Haas et al., 2002; Gong et al., 2007; Kobow et al., 2009; Haas and Frotscher, 2010; Freiman et al., 2011). Loss of the reelin adaptor protein, disabled-1 (Dab1), imparts a similar developmental phenotype to loss of reelin function (Howell et al., 1997a; Sheldon et al., 1997; Olson et al., 2006). In prior work, we conditionally deleted Dab1 from nestin-expressing neural stem cells (NSCs) in the postnatal mouse subgranular zone (SGZ) and found that NSCs deficient in Dab1 generate adult-born neurons that migrate ectopically and have abnormal dendritic development (Teixeira et al., 2012). Here we explore whether the disrupted integration of Dab1-deficient early postnatal- through adult-born neurons leads to altered seizure susceptibility or epilepsy.

## Materials and methods

### Animals and tamoxifen administration

All procedures and experiments were approved by the University of Michigan Institutional Committee on the Use and Care of Animals and were performed in accordance with guidelines developed by the National Institutes of Health. NestinCreER^*T*2^/R26RYFP/Dab1^*Flox*/*Flox*^ triple transgenic mice were derived and maintained as previously described (Teixeira et al., 2012). Briefly, mice harboring the Dab1-conditional allele (Pramatarova et al., 2008) were crossed with *Nestin-Cre-ER*^*T*2^ mice (Lagace et al., 2007; “Line K” in Sun et al., 2014) and a recombination reporter line, R26R-YFP, containing a floxed-stop followed by yellow fluorescent protein (YFP) in the *ROSA26* gene locus (Zambrowicz et al., 1997). Thus, tamoxifen (TMX)-inducible, cre-mediated recombination results in the deletion of Dab1 along with expression of YFP in nestin-expressing progenitors and their subsequent progeny. Bigenic mice with wild-type Dab1 treated with TMX and mice with mutant Dab1 but not treated with TMX were used as controls. Heterozygous mice (NestinCreER^T2^/R26RYFP/Dab1^*Flox*/+^) served as a gene dose comparison to the homozygous null mutants. We refer to homozygous mutants as Dab1^*Flox*/*Flox*^, heterozygous as Dab1^*Flox*/+^, and controls as Dab1^+/+^. Experimenters were blind to genotype and pups were treated i.p. once daily with TMX at 100 mg/kg (dissolved in 10% EtOH/90% sunflower oil, 12.5 mg/ml) or vehicle on P7 and P8. Animals survived to P55 before any additional manipulations were performed (Supplemental Figure 1). Unless otherwise noted, mice were housed under a 12 h light/dark cycle and were given food and water *ad libitum*.

### Epidural electrode placement and Video-EEG

To determine if Dab1-deficient mice exhibit spontaneous seizures or epileptiform activity, we monitored animals with continuous video-EEG. Mice were implanted with four epidural screw electrodes at 2 months after the last administration of TMX. Procedures for affixing electrodes were performed as previously described (Lee et al., 2012; Kehrl et al., 2014; Wagnon et al., 2015). Mice were anesthetized with ketamine and xylazine and placed in a stereotaxic mouse adaptor (Stoelting). Six burr holes were made in the skull using a 3.2 mm steel bit (Meisinger USA, Centennial, Colorado). Electrodes were positioned and fastened (left and right frontal, left and right parietal, one cerebellar, and one reference over the sinus cavity) using mounting screws (E363/20; PlasticsOne, Roanoke, VA). The sockets were fitted into a 6-pin electrode pedestal and the entire apparatus was secured with dental cement (Stoelting). Animals received buprenorphine (0.05 mg/kg) every 12 h for 3 days after surgery. Following recovery, animals were monitored continuously for 10 days by video/EEG recording (Ceegraph Vision; Natus, Middleton, WI). Recordings were sampled at 256 Hz and concurrent video was analyzed offline and synced with EEG data in Persyst 12 (Persyst, Prescott, AZ). Seizures and epileptiform activity were assessed manually in their entirety by an observer blind to the genotypes. Interictal epileptiform discharges (IEDs) were defined as paroxysmal waveforms lasting 20–200 ms having spike or sharp-wave and after-going slow wave morphology, identified visually to be independent of preceding behavioral activity. Spontaneous seizures were defined as the abrupt appearance of rhythmic waveforms or runs of spikes/sharp waves that persisted for a minimum of 10 s and displayed an unequivocal evolution in frequency and morphology, followed by postictal suppression and return to baseline. Detailed evaluation of EEG waveforms was performed by randomly selecting a 4-h epoch from each day during the 10 day recording period.

### Pilocarpine administration and seizure threshold

Mice were injected intraperitoneally with scopolamine methyl nitrate (5 mg/kg) to block peripheral cholinergic effects, followed 20 min later with pilocarpine hydrochloride (289 mg/kg), a muscarinic cholinergic agonist, to produce SE. Animals were monitored for 45 min and were given additional half-doses (not exceeding twice the original dose) of pilocarpine if no seizure activity was detected.

The clinical manifestation of SE was scored using a modified Racine scale (Racine, 1972). The scoring was: 0 for mice displaying no abnormal behavior; 1 for periods of freezing, chewing or nodding; 2–3 if tremors and shaking developed, along with straub tail; 4 for mice with seizures that included rearing or clonus, followed by a clear recovery; 5 was used for mice with rearing and clonus, along with running and falling; 6 for severe and repeated stage 5 seizures where animals either did not recover between events or expired.

Seizure threshold was defined as the latency (in minutes) from the administration of pilocarpine until the animal experienced the electrographic onset of SE, consisting of either a prolonged seizure lasting at least 3 min or 3 contiguous seizure events less than 2-min apart from one another without intervening complete behavioral recovery. Electrographic onset was defined by a distinctive shift in background activity whereby low-amplitude high frequency waves evolved into large, rhythmic epileptiform discharges, at least one standard deviation greater in amplitude compared to background activity.

### Tissue processing and immunohistochemistry

Immediately after a 90-min SE episode, mice were injected with an overdose of sodium pentobarbital and perfused intracardially with 0.9% saline followed by 4% paraformaldehyde (PFA) in phosphate buffered saline (PBS), pH 7.4. Brains were fixed overnight, cryoprotected in 30% sucrose at 4°C, and cyrosectioned at 40 μm in the coronal plane using a Leica CM1850 cyrostat (Leica Microsystems, St. Louis, MO). Sections were collected into cyropreservative (sucrose and ethylene glycol in 50 mM phosphate buffer) as series of every 12th section through the entire extent of the septotemporal axis and stored at −20°C until processed for immunohistoschemistry (IHC). Tissues were rinsed thoroughly in Tris buffered saline (TBS, pH 7.6) and incubated for 90 min at room temperature in a blocking solution containing 10% normal goat serum, triton x-100, glycine, and bovine serum albumin in TBS. Floating sections were incubated overnight at 4°C with a cell type-specific antibody along with a chicken anti-GFP (1:2000, Aves Labs - GFP-1020; Cho et al., 2015) to enhance the endogenous YFP fluorescence and confirm colocalization. Cell-type specific antibodies included rabbit anti-doublecortin (DCX, 1:1000, AbCam - ab18723; Lee and Umemori, 2013), mouse anti-NeuN (1:2000, Chemicon - MAB377; Miltiadous et al., 2013), and rabbit anti-Prox1 (1:2000, Millipore - AB5475; Farrar et al., 2005; Parent et al., 2006). We verified deletion of Dab1 by using a rabbit anti-Dab1 antibody (1:1000, Sigma − ab-232; Howell et al., 1997b; Teixeira et al., 2012 and **Figures 3A,B'**). Rabbit anti-MAP2 (2a+2b) antibody (1:1000, Sigma-M2320; Quadrato et al., 2014) was used to visualize hippocampal dendritic organization. Rabbit Ki67 antibody was used to label actively dividing cells (1:1000, Vector Labs–VP-K451; Singer et al., 2009). Alexa secondary antibodies (1:600; ThermoFisher) were used for single or double immunofluorescence. In some instances tissue was briefly rinsed with Hoescht 33342 (1:5000) for a bisbenzimide nuclear counterstain. Low-magnification images were acquired on a Leica DMI 6000 inverted scope and higher magnification images for co-localization were collected as thin optical section (1–2 μm) z-stacks on a Nikon A-1 laser scanning confocal microscope.

### Data quantification

For colocalization with each cell type-specific antigen, we quantified the left or right hemisphere of 4–5 sections per animal. Three-dimensional stacks were viewed as a maximum intensity projection and a grid comprised of 100 × 100 μm squares was placed over the image in ImageJ 1.48 (NIH, Bethesda, MD). Squares containing the GCL and SGZ were numbered and at least three were selected at random for quantification. Cells expressing the reporter were identified in the z-stack and colocalization of the Alexa secondary was confirmed in x and y dimension using Nikon Element Viewer (Melville, NY). No fewer than 5 cells were counted per section and a minimum of 30 cells were counted per animal. Values are reported as cells that express YFP and cell type–specific antigen over total number of YFP-positive cells. For recombination efficiency, we estimated the number of YFP-labeled cells per 100 μm^2^ by averaging the number of cells over three tissue sections for each animal. We verified localization of each cell type-specific antigen with bisbenzimide. Density of DCX+ cells was estimated with the aid of MicroBrightField Neurolucida (MBF, Williston, VT). For each animal, we selected a 500 μm linear region along the GCL and included cells within the SGZ and GCL. Only whole round or spherical cell bodies were counted and fragments of DCX labeling less than 5 μm were excluded. Cells were tagged with a digital marker through each optical section to prevent double-counting. Clusters of cells where one cell body merged into another were counted as one, thus our quantification underestimates the total number of DCX-positive cells. This number is reported as average number of DCX-expressing neurons per 500 μm. The density of hilar ectopic granule cells (HEGCs) was determined by counting all Prox1+ cells within a defined region of interest restricted to the hilar region of the DG, delineated as 20 μm from the bottom of the GCL. Representative images of labeling were exported as TIFF files and edited using Corel PaintShop Pro X7 (Corel, Ottawa, Ontario, Canada). Images were cropped to fit the dimensions of each figure.

### Statistical analyses

All statistical analyses were made using JMP Pro 11 software (SAS, Cary, NC). Data sets were checked for normal distributions. ANOVA with *post-hoc* test was performed to assess differences across multiple comparisons. Individual comparisons were made using Tukey's HSD test to correct for sample number. We performed a non-parametric comparison using Wilcoxon's *t*-test for comparison between groups for latency and Spearman's ρ or Fisher's Exact Test (small sample sizes) for multivariate correlations. Values are reported as mean ± SEM, for all statistical comparisons: ^*^p < 0.05, ^**^p < 0.02.

## Results

### Dab1–deficient adult mice exhibit infrequent interictal EEG abnormalities and rare seizure-like events

**Figure 1:**
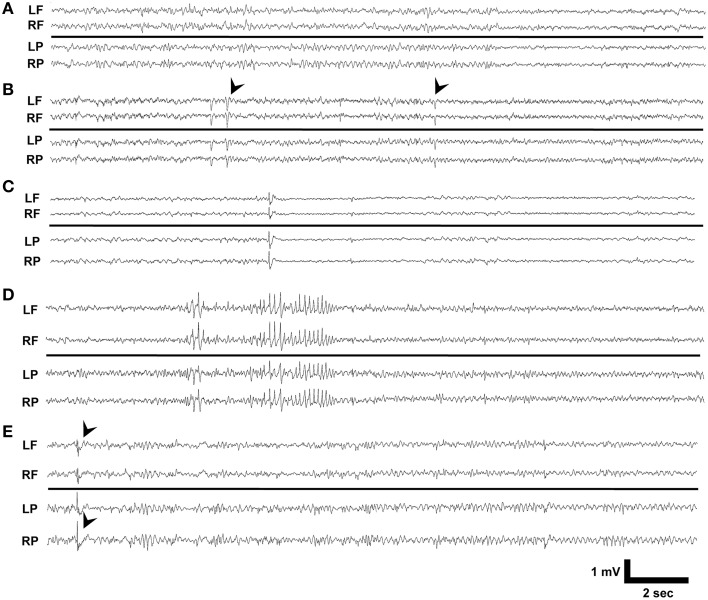
**EEG phenotypes of Dab1^***Flox***/***Flox***^ mutants**. **(A)** Typical interictal EEG which exhibits no abnormalities, interictal spikes, or epileptiform discharges. **(B)** Intermittent sharp waves and background slowing which occasionally was associated with a myoclonic spasm (black arrowheads). This patterned activity persisted for 3 min. **(C)** Prolonged periods of low amplitude beta with intermittent spikes coinciding with behavioral inactivity. **(D)** High amplitude, sharply contoured theta waveforms that preceded myoclonus. **(E)** Interictal spike discharges (black arrowheads) appeared throughout the 10 day recording period as the sole abnormality in this mouse. LF, left frontal; RF, right frontal; LP, left parietal; RP, right parietal.

We reviewed 10 day video-EEG records and found that no vehicle treated Dab1^*Flox*/*Flox*^ (*n* = 10), vehicle or TMX treated Dab1^*Flox*/+^ (*n* = 5) or TMX treated Dab1^+/+^ (*n* = 9), exhibited seizures or IEDs (Figure 1A). A total of 3 of the 17 Dab1^*Flox*/*Flox*^ mice that received TMX exhibited IEDs. One mouse exhibited brief myoclonic spasms, with preceding spike-wave discharges (black arrowheads, Figure 1B). We identified a second animal (Figure 1C) with recurrent epileptiform discharges at regular intervals with background suppression and slowing, coinciding with prolonged periods of immobility, tremors, and labored movement. A third mouse (Figure 1D) displayed intermittent 2–4 s discharges of large amplitude theta waves that were occasionally followed by brief spasms, not associated with an obvious epileptiform EEG discharge. Lastly, a single mouse exhibited intermittent spike-wave discharges (black arrowheads, Figure 1E) throughout the 10-day recording period but experienced no behavioral events. These observations indicate that deletion of Dab1 in postnatal NSCs does not cause overt spontaneous convulsive seizures, but leads to hyperexcitable network changes that can generate spike discharges and sporadic EEG patterns with behavioral events on an interictal-to-ictal continuum.

### Adult mice deficient in Dab1 exhibit more severe seizures and a reduced latency to the onset of pilocarpine-induced SE

To probe whether postnatal Dab1 deletion in DGCs alters susceptibility to developing SE after pilocarpine administration, we injected drug while animals were still being monitored by video-EEG. In addition to scoring the severity of the seizures, we measured the latency to onset of the first seizure and to the beginning of SE.

**Figure 2:**
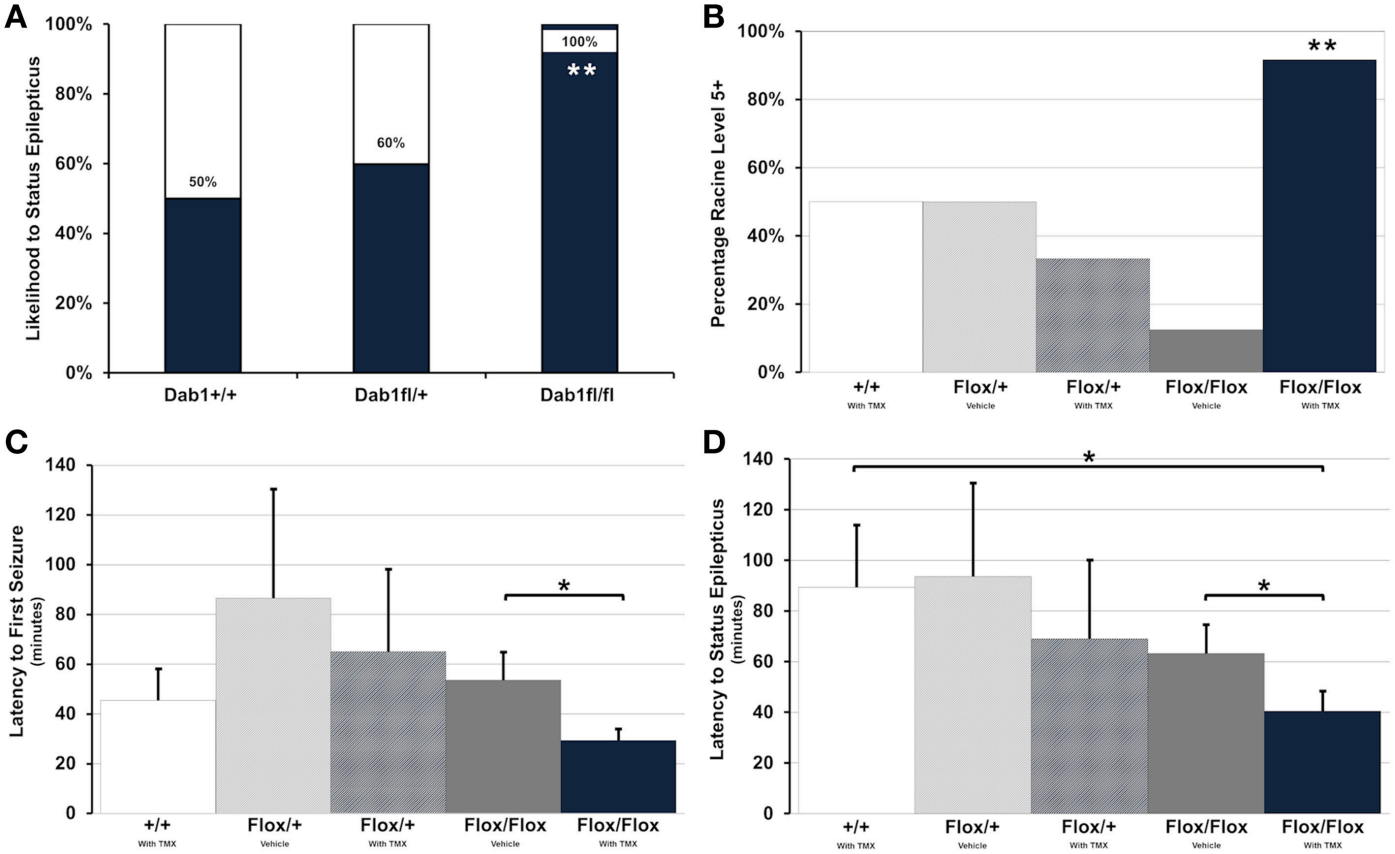
**Conditional deletion of Dab1 reduces SE latency and increases seizure severity. (A)** Dab1^*Flox*/*Flox*^ mice were significantly more likely to develop SE compared to Dab1^*Flox*/+^ or Dab1^+/+^ mice. **(B)** Compared to all other treatment groups, TMX treated Dab1^*Flox*/*Flox*^ mice were significantly more likely to develop severe, Racine stage 5 or 6 seizures following the administration of pilocarpine. **(C)** TMX-treated Dab1^*Flox*/*Flox*^ mice exhibited the shortest latency to the first seizure and **(D)** a significantly shorter latency to SE compared to vehicle treated Dab1^*Flox*/*Flox*^ or Dab1^+/+^mice. For all statistical comparisons: ^*^p < 0.05, ^**^p < 0.02.

All Dab1^*Flox*/*Flox*^ mice, regardless of whether or not they were treated with TMX, developed SE (Figure 2A, *n* = 20). This was in contrast to only 50% of Dab1^+/+^ (*n* = 4) and 67% of Dab1^*Flox*/+^ (*n* = 5) mice, indicating that mutant mice are significantly more likely to develop SE (Fisher's Exact Test; *p* < 0.02). Mutants typically required less pilocarpine to develop SE, but we determined that this variability was not significant (Fisher's Exact Test; *p* = 0.27). In some cases, mice that failed to develop SE exhibited brief seizures. Of the animals that did develop behavioral and electrographic SE, only mutants that received TMX (*n* = 12) were likely to develop severe, stage 5 seizures (Figure 2B, Fisher's Exact Test; *p* < 0.02). Only three mice expired during the course of the experiments (1 each in Dab1^+/+^ + TMX, Dab1^*Flox*/*Flox*^ + vehicle, and Dab1^*Flox*/*Flox*^ + TMX groups), and mortality did not differ between groups (Fisher's Exact Test; *p* = 0.86).

Dab1^*Flox*/*Flox*^ mice treated with TMX consistently had the earliest onset to the first seizure (Figure 2C), which was significantly shorter compared to Dab1^*Flox*/*Flox*^ given vehicle (Dab1^*Flox*/*Flox*^ + TMX: *n* = 12, 29.4 ± 4.1; Dab1^*Flox*/*Flox*^ + vehicle: *n* = 8, 53.6 ± 10.7; *p* < 0.05). Dab1^*Flox*/*Flox*^ mice had a significantly reduced latency to SE (Figure 2D, 40.5 ± 7.4) compared to Dab1^*Flox*/*Flox*^ animals treated with vehicle (63.3 ± 10.7; *p* < 0.05) and mice with WT Dab1 (89.3 ± 24.2; *p* < 0.05), but were not different than Dab1^*Flox*/+^ mice given TMX (69.0 ± 30.6; *p* = 0.17) or vehicle (93.5 ± 36.5; *p* = 0.08). From these data we conclude that mice with conditional Dab1 deletion in postnatal DGCs develop seizures that evolve into SE more quickly than controls, and exhibit a greater seizure severity after pilocarpine treatment.

### Recombination efficiency and deletion of Dab1 is negatively correlated with latency to SE

**Figure 3:**
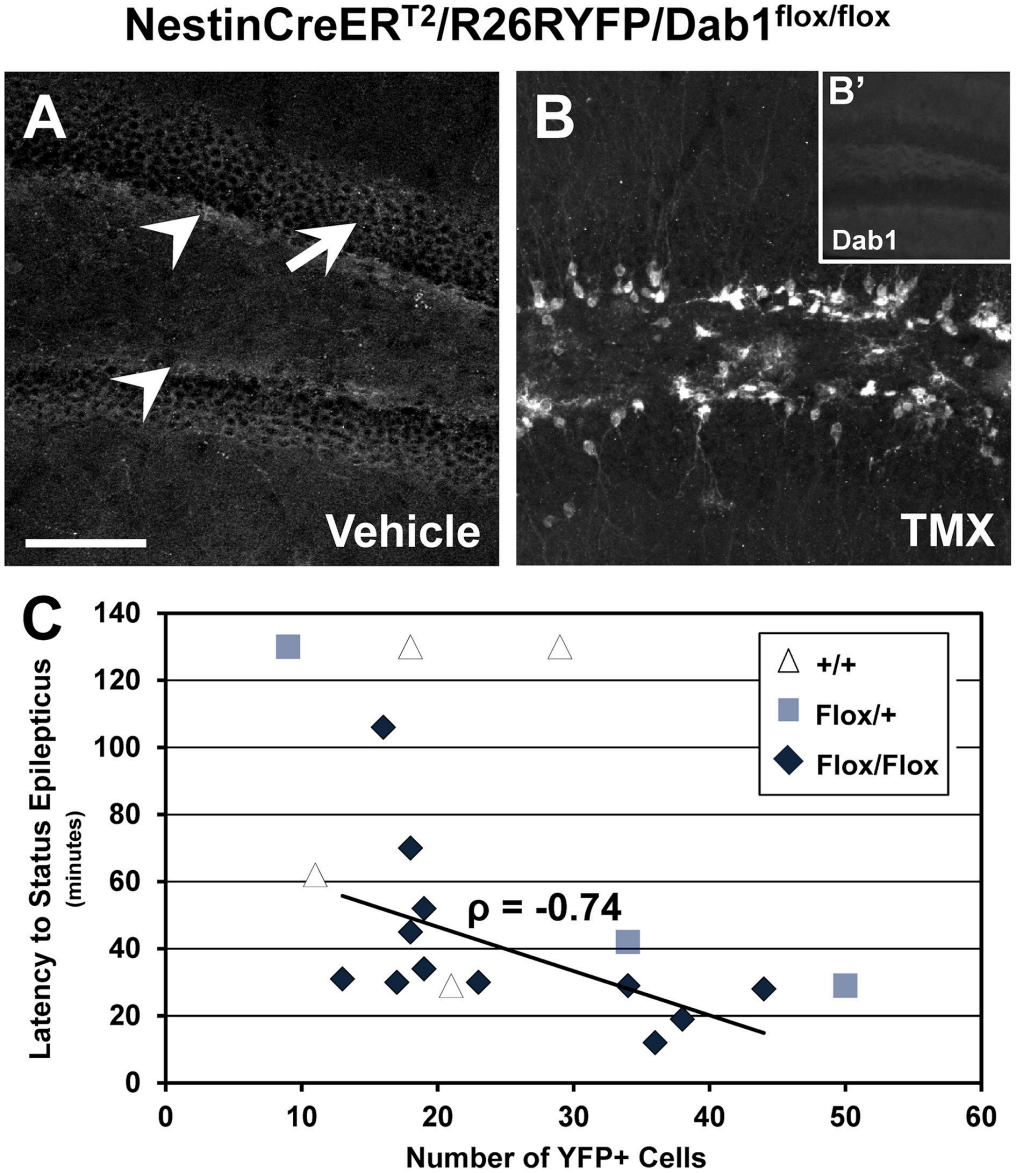
**Recombination efficiency and latency to status epilepticus in NestinCreER^**T2**^/R26RYFP/Dab1^***Flox***/***Flox***^ mice. (A–B′)** Confocal images of sections through the DG immunostained for Dab1 **(A,B**′**)** or YFP **(B). (A)** Dab1 expression is distributed through the SGZ (arrowheads) of the DG in untreated Dab1^*Flox*/*Flox*^ mice. Scattered labeling is present in the GCL (arrow) and to a lesser extent within the molecular layer and hilus. **(B)** Two days of TMX treatment induces Cre activation and recombination in Dab1^*Flox*/*Flox*^ mice, resulting in substantial YFP reporter expression 2 months later. **(B**′**)** Note that virtually no Dab1 expression remains in the DG after TMX treatment. **(C)** There is a significant inverse correlation between recombination efficiency and latency to SE in TMX treated Dab1^*Flox*/*Flox*^ mice, indicating that the latency to SE is influenced by reelin signaling in postnatal- or adult-born DGCs. No correlation exists for Dab1^+/+^ mice. Scale bar = 100 μm **(A,B)**, 250 μm in **(B**′**)**.

Dab1 protein is predominately expressed throughout the SGZ of adult mice (white arrowheads, Figure 3A), and at lower levels in the GCL (white arrow, Figure 3A). Sporadic Dab1-positive neurons were also seen in the hilus and inner molecular layer. Treatment with TMX resulted in the expression of YFP (Figure 3B) and loss in the expression of virtually all Dab1 protein (Figure 3B′). YFP expression was limited to regions that exhibit postnatal neurogenesis.

Using reporter activity as a proxy for recombination efficiency, we sought to determine if latency to SE varied as a function of the loss of Dab1 protein (Figure 3C). We found a significant inverse correlation between recombination efficiency after TMX treatment and SE onset latency for Dab1^*Flox*/*Flox*^ (*n* = 12; ρ = −0.74, *p* < 0.02), an inverse correlation for Dab1^*Flox*/+^ (*n* = 3; ρ = −1), but not Dab1^+/+^ (*n* = 4; ρ = 0.41) mice. This finding suggests that progressively greater loss of Dab1 results in a more pronounced susceptibility to pilocarpine-induced SE. Thus, despite the absence of definite spontaneous seizures, altered circuitry associated with the conditional deletion of Dab1 appears to be pro-epileptic.

### Dab1^*Flox*/*Flox*^ mice exhibit a dispersed CA1 pyramidal cell layer which does not correlate with seizure phenotype

**Figure 4:**
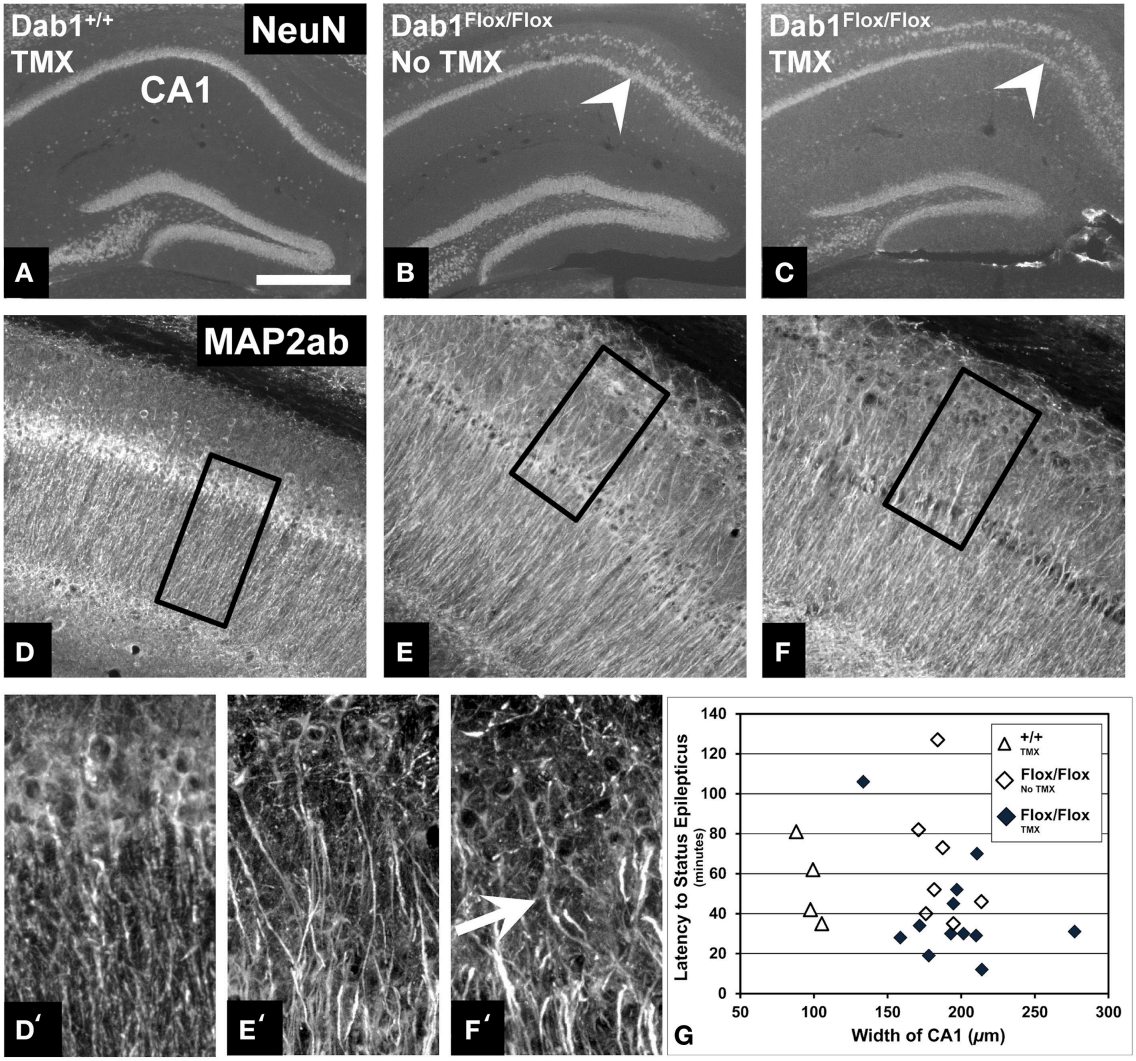
**Abnormalities of the CA1 pyramidal layer do not correlate with latency to SE. (A)** Labeling for NeuN demonstrates that the CA1 pyramidal cell layer of mice with WT Dab1 is single layered compared to the ectopic layer (above the white arrowheads, which point to the “normal” layer) found in hypomorphic mice harboring floxed-Dab1, regardless if they are treated with vehicle **(B)** or TMX **(C)**. Similar to mice with WT Dab1 **(D)**, the primary pyramidal layer of Dab1^*Flox*/*Flox*^ mice treated with vehicle **(E)** or TMX **(F)**, exhibit intact apical dendrites. **(D**′**)** Dendrites from pyramidal neurons of WT mice extend perpendicular to the long access of the cell layer. In contrast, dendrites of the ectopic pyramidal layer in mice with Floxed-Dab1, treated with vehicle **(E**′**)** or TMX **(F**′**)** have curved apical arbors (white arrow) with overlapping domains. **(D**′**–F**′**)** are the boxed regions in **(D–F)**, respectively. **(G)** Despite this pronounced phenotype, there was no correlation between overall width of the CA1 pyramidal layer and latency to SE. Scale bar = 500 μm **(A–C)**, 300 μm **(D–F)**, and 50 μm **(D**′**–F')**.

We next sought to examine structural alternations potentially underlying the increased seizure susceptibility caused by conditional Dab1 deletion in postnatal NSCs and their progeny. Mice homozygous for floxed *Dab1* have reduced Dab1 protein levels from P1-7, even without cre driven recombination (Pramatarova et al., 2008; Teixeira et al., 2014). This coincides with the period of development when pyramidal neurons migrate away from the ventricular zone and the hippocampal primordium to stratify in the outer lamina of CA1 (Stanfield and Cowan, 1979; Nakahira and Yuasa, 2005). It is not surprising then that the CA1 pyramidal layer is disrupted in mutants whether treated with vehicle or TMX (Figures 4A–C). MAP2ab labeling, which allows for visualization of CA1 apical dendrites, revealed that the ectopic layer of pyramidal neurons maintain their apical arbors (Figures 4D–F). In contrast to the highly organized dendritic fields of the inner layer, dendrites emanating from ectopic pyramidal neurons were notably tortuous (white arrow in Figure 4F′). This phenotype did not appear to contribute to the reduced seizure latency as we found no correlation between the mean width of the CA1 layer and latency to SE (Figure 4G) in vehicle (ρ = −0.36, *p* = 0.43) or TMX treated (ρ = −0.14, *p* = 0.66) mice, regardless of genotype (data not shown).

### Dab1-deficient NSCs produce fewer immature neurons but more HEGCs, the degree of which correlates with reduced seizure latency

Previous work suggests that loss of Dab1 in postnatal NSCs and their progeny alters DGC development and maturation (Teixeira et al., 2012). To assess the degree of neurogenesis and location of DGCs after conditional Dab1 deletion, we performed IHC to identify proliferating cells (Ki67), immature neurons (DCX), and post-mitotic granule cells (Prox1).

**Figure 5:**
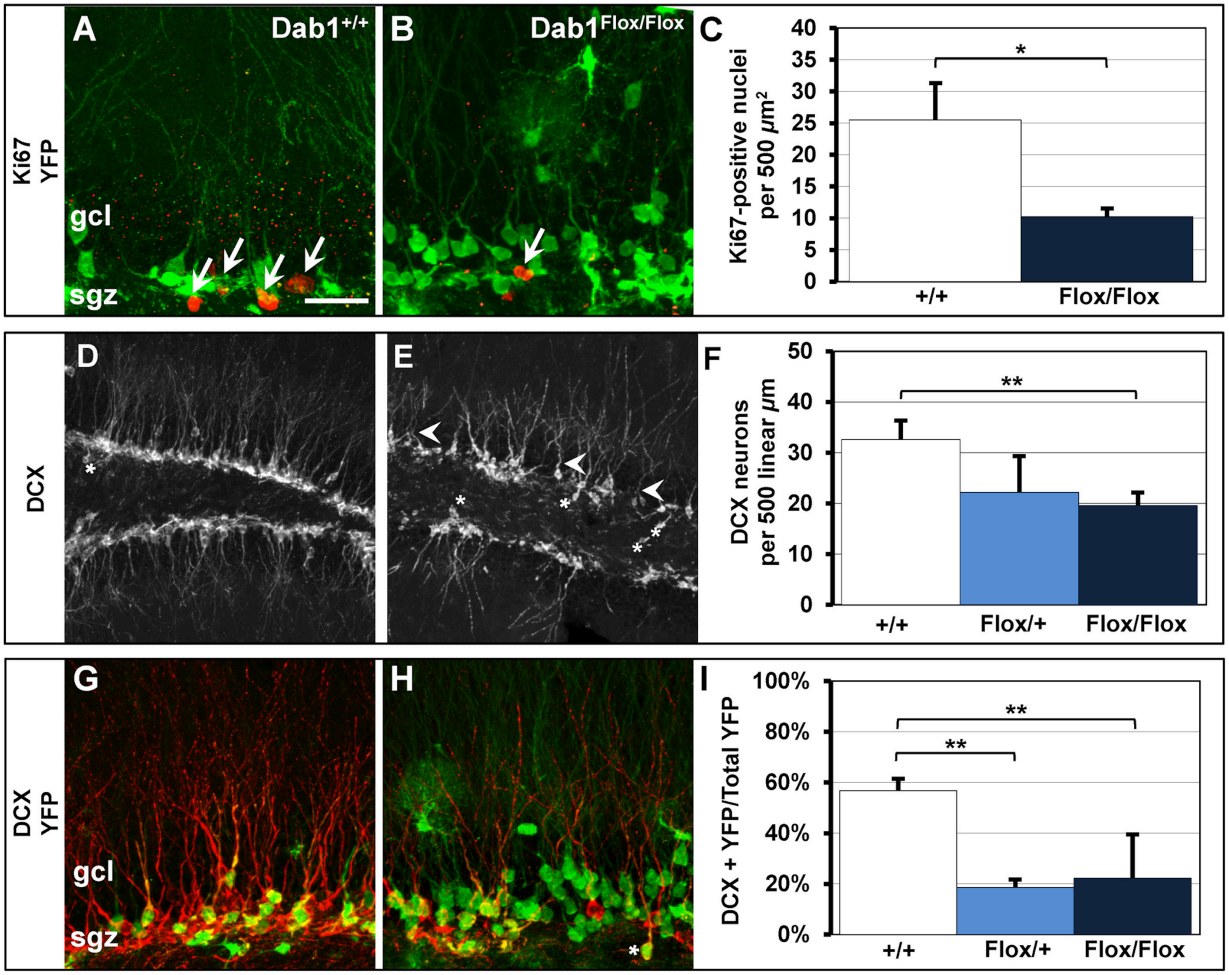
**Cell proliferation and DCX-positive immature neurons are reduced in Dab1-deficient mice. (A)** Ki67-labeled nuclei are distributed throughout the SGZ of Dab1^+/+^ mice treated with TMX and are often seen in clusters (white arrows). **(B)** In contrast, sparsely distributed Ki67-labeled nuclei (white arrow) are observed in Dab1^*Flox*/*Flox*^ mice treated with TMX. **(C)** There are significantly fewer Ki67-labeled nuclei in Dab1^*Flox*/*Flox*^ mice treated with TMX compared to controls treated with TMX. **(D)** DCX-expressing immature neurons are distributed throughout the SGZ and inner GCL of WT mice. Most have distinct apical dendrites (white arrowheads) and very few are found in the hilus (asterisk). **(E)** In contrast, we observe a sparse distribution of DCX-positive immature neurons in Dab1-deificient mice. Immature neurons exhibit fewer, atrophic, apical dendrites (white arrowheads) and several are located ectopically (asterisks). **(F)** There are significantly fewer DCX-expressing immature neurons throughout the SGZ and GCL of Dab1^*Flox*/*Flox*^ mice treated with TMX compared to controls, but not Dab1^*Flox*/+^ mice, treated with TMX. **(G)** DCX/YFP co-expressing immature neurons are distributed throughout the SGZ and inner third of the granule cell layer. **(H)** Despite the appearance of more YFP labeling, fewer YFP+ cells co-localized with DCX throughout the SGZ, and some appeared ectopic (asterisks). **(I)** Significantly fewer DCX/YFP double-labeled cells appeared in Dab1^*Flox*/+^ and Dab1^*Flox*/*Flox*^ mice compared to controls. Scale bar (in **A**) = 20 μm for **(A**,**B**,**G**,**H)** and 100 μm for **(D,E)**.

We found significantly fewer actively proliferating cells in Dab1^*Flox*/*Flox*^ mice (Figure 5A, *n* = 6; 10.3 ± 1.0) compared to controls (Figures 5B,C; *n* = 5; 25.5 ± 5.5; *p* < 0.02). Consistent with the decreased cell proliferation, there were also less DCX-labeled immature neurons observed in TMX treated Dab1^*Flox*/*Flox*^ mice compared to wild-type vehicle treated controls (Figures 5D,E), suggesting decreased neurogenesis. As with our previous findings (Teixeira et al., 2012), immature neurons exhibited stunted dendrites with atrophic apical branches (white arrowheads, Figure 5E). We found fewer DCX-positive immature neurons in TMX treated Dab1^*Flox*/*Flox*^ mice (Figure 5F; *n* = 14; 19.6 ± 2.2 per 500 linear μm) compared to TMX treated Dab1^+/+^ mice (*n* = 8; 32.6 ± 3.4; *p* < 0.02*)*, but not Dab1^*Flox*/+^ mice (*n* = 3; 22.2 ± 6.7; *p* = 0.10).

We then quantified the percentage of DCX-positive cells that co-expressed YFP to determine how loss of Dab1 directly affects neuronal differentiation (Figures 5G,H). We observed significantly fewer DCX+/YFP+ immature neurons in TMX-treated Dab1^*Flox*/*Flox*^ (Figure 5I; *n* = 17; 22.3% ± 0.03) and Dab1^*Flox*/+^ mice (*n* = 3; 18.6% ± 0.04) compared to TMX-treated controls (*n* = 9; 56.8% ± 0.05, *p* < 0.02). From these data we conclude that the loss of Dab1 reduces the proliferation of DG neural progenitors, which contributes to the decrease in the number of immature neurons.

**Figure 6:**
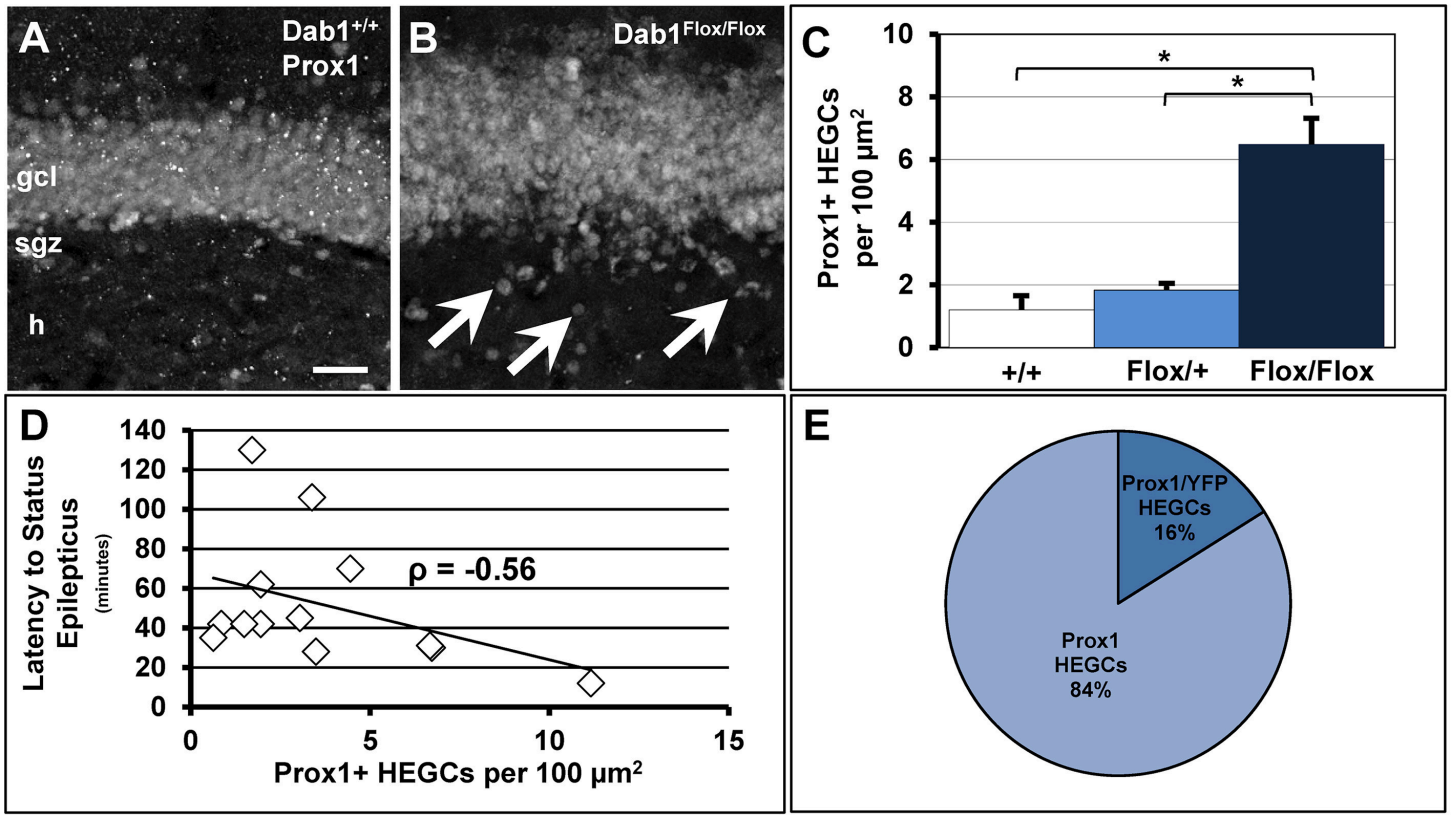
**Conditional deletion of Dab1 results in non-cell autonomous ectopic migration of Prox1+ granule cells that inversely correlates with latency to SE**. **(A)** The GCL of Dab1^+/+^ mice given TMX is compact with very few Prox1+ cells found outside the SGZ or GCL. **(B)** The overall structure of the GCL is disrupted in TMX-treated Dab1^*Flox*/*Flox*^ mice, with many Prox1+ HEGCs evident (arrowheads). **(C)** The density of HEGCs in Dab1^*Flox*/*Flox*^ mice was significantly greater compared to controls and Dab1^*Flox*/+^ mice after TMX treatment. ^*^p < 0.05. **(D)** The density of Prox1 + HEGCs inversely correlated with latency to SE. **(E)** The majority of Prox1 + HEGCs were negative for YFP, supporting a non-cell autonomous effect of the loss of reelin signaling. Scale bar = 50 μm in **(A,B)**.

We previously demonstrated that adult-born neurons generated from Dab1-deficient, nestin-expressing NSCs and their progeny migrate ectopically (Teixeira et al., 2012). Here we confirm the observation that mice deficient for Dab1 exhibit significantly more Prox1+ HEGCs (Figures 6A–C; *n* = 12, 6.48 ± 0.75 per 100 μm^2^) than TMX-treated controls (*n* = 6, 1.20 ± 0.36 per 100 μm^2^, *p* < 0.02) or Dab1^*Flox*/+^ mice (*n* = 2, 1.83 ± 0.12 per 100 μm^2^, *p* < 0.05). Notably, the number of HEGCs inversely correlated with the latency to onset of SE (Figure 6D; *n* = 13, ρ = −0.56, *p* < 0.05). Initially, we reasoned that it was the cell autonomous deletion of Dab1 from nestin-expressing NSCs that was responsible for causing Dab1-deficient neurons to migrate ectopically away from the GCL. In examining YFP+/Prox1+ granule cells (data not shown), we noted that the majority of HEGCs were negative for YFP reporter expression (Figure 6E, 83.95% ± 0.03). Taken together, the conditional deletion of Dab1 from postnatal nestin-expressing NSCs results in a non-cell autonomous ectopic migration of granule cells into the hilus, and their presence correlates with a reduced latency to SE onset.

## Discussion

We sought to determine if changes that arise following conditional deletion of Dab1 from postnatal NSCs and their progeny cause spontaneous seizures. We observed no definite behavioral or electrographic seizures, but some mice experienced single myoclonic spasms associated with EEG discharges, and others showed three different patterns of epileptiform or paroxysmal discharges among 4 of 17 TMX-treated mutant mice. These findings suggest that the conditional loss of reelin signaling in postnatally-generated DGCs does not result in a robust epilepsy phenotype. However, we did find that TMX-treated Dab1^*Flox*/*Flox*^ mice are more likely to develop severe SE after administration of the chemoconvulstant pilocarpine, with a shorter latency than TMX-treated heterozygous or wild-type mice, as well as vehicle-treated homozygous littermates. Histological analyses confirmed that the postnatal loss of Dab1 in NSCs induces the ectopic migration of DGCs (Teixeira et al., 2012) and reduces ongoing adult neurogenesis. Possible mechanisms for this latter finding may involve promoting NSC quiescence, changing the phenotype of NSC progeny from neuronal to glial (Teixeira et al., 2012), or decreasing the proliferation or survival of transit amplifying progenitors or immature neurons.

### The development of HEGCs in Dab1^*Flox*/*Flox*^ mice is not sufficient to generate spontaneous seizures

Among the more notable changes in Dab1^*Flox*/*Flox*^ mice is the ectopic placement of adult-born granule cells into the hilus and along the border between the GCL and the inner molecular layer of the dentate gyrus (Figure 6; Teixeira et al., 2012). Dispersion of the GCL and HEGCs are hallmark features in humans with mTLE (Lurton et al., 1998; Parent et al., 2006; Blumcke et al., 2009; Bae et al., 2010) and can be reproduced in models that induce SE with kainic acid, pilocarpine, or limbic kindling (Bouilleret et al., 1999; McCloskey et al., 2006; Botterill et al., 2015). Several studies have confirmed that the loss of reelin correlates with aberrant migration (Haas et al., 2002; Heinrich et al., 2006; Gong et al., 2007; Haas and Frotscher, 2010), but our study is unique in several ways. In contrast to the overt developmental phenotype of the *reeler* mutant mouse, conditional deletion of Dab1 from postnatal nestin-expressing NSCs allows us to explore whether the aberrant development of neonatal- and adult-born neurons disrupt an already established circuit. Secondly, our approach evaluates the contribution of displaced neonatal- or adult-born DGCs to a potential seizure phenotype, separate from the ectopic dispersion of mature DGCs in the DG (Heinrich et al., 2006). Another striking observation is the large percentage of ectopic Prox1+/YFP- DGCs, presumably those expressing normal levels of Dab1. Based upon this finding, we postulate that although the loss of Dab1 in the progeny of NSCs may contribute to a migration deficit, the majority of HEGCs migrate into the hilus due to a non-cell autonomous effect of postnatal Dab1 deletion.

A prevailing hypothesis is that HEGCs contribute to the development of spontaneous seizures and epilepsy (Scharfman and Pierce, 2012), but our observation that mice with ectopically located granule cells are largely seizure-free suggests that there are unaccounted for factors that promote seizure development. Our findings are similar to the study by Koyama et al. (2012) where they demonstrated that generating aberrantly located neurons without inducing seizures was not sufficient to produce rats with epilepsy. Deletion of the pro-apoptotic gene *BAX* generates mice with HEGCs, and though the mice are deficient in a pattern separation task, they also remain seizure-free (Myers et al., 2013). These observations, along with the current study, challenge the hypothesis that HEGCs are sufficient for driving spontaneous seizures.

This does not eliminate the possibility that HEGCs can act as “hub” cells within a seizure network (Morgan and Soltesz, 2008; Cameron et al., 2011). There is clear electrophysiological evidence that they are hyperexcitable and make aberrant connections (Scharfman et al., 2000; Zhan and Nadler, 2009; Althaus et al., 2015). There is also strong correlative evidence between the presence of HEGCs and seizure severity (McCloskey et al., 2006; Hester and Danzer, 2013). It is possible that our deletion is not sufficient enough to generate enough aberrantly placed GCs. In support of this idea, the mouse with the highest recombination efficiency and that also had the greatest density of HEGCs did not show abnormalities on video/EEG monitoring. However, this mouse did exhibit the shortest latency to SE. We also considered the possibility that we did not allow enough time for the aberrant neurons to integrate. We addressed this by extending the period after TMX to 4 months, but these mice were also seizure-free. Though we cannot definitively exclude the possibility that there are subcortical seizures with a limited focal onset, HEGCs are not sufficient to cause epilepsy with clinical manifestations via cortical EEG, including generalized tonic-clonic seizures.

Notably, the study showing spontaneous seizure development after postnatal deletion of PTEN in murine DGCs also demonstrated substantial structural abnormalities in PTEN-deficient HEGCs and those in the GCL. These changes included increased soma size and an excess of dendritic spines, features that are likely important for the development of spontaneous seizures (Pun et al., 2012). We did not observe such structural abnormalities after postnatal Dab1 deletion. In summary, our findings suggest that HEGCs produce network instability, but additional aberrant structural or functional features are necessary to cause epilepsy.

### CA1 pyramidal cell layer disruption in Dab1 hypomorphs does not contribute to hyperexcitability in the hippocampus

Mice expressing floxed *dab1* alleles, whether they are treated with vehicle or TMX, have been shown to express less protein during early postnatal development than the wild-type, and this hypomorphic effect causes abnormalities in the cerebellum and CA1, but not the dentate gyrus (Herrick and Cooper, 2002; Matsuki et al., 2008; Pramatarova et al., 2008). More recently, this structural phenotype was implicated in causing deficits in maternal behavior and spatial learning (Teixeira et al., 2014). Other genetic models that exhibit a similar heterotopia of CA1 exhibit deficits in long-term potentiation (Petrone et al., 2003). Occasionally, a bilayered CA1 pyramidal cell layer is even present in human mTLE hippocampal specimens (Sloviter et al., 2004). All of these observations are consistent with a role of this morphological abnormality in mTLE or its associated comorbidities. We noted that the CA1 phenotype was particularly severe in our Dab1^*Flox*/*Flox*^ mice (Figures 4A–C), raising concerns that the ectopic CA1 may contribute to overall hyperexcitability. The strongest evidence to the contrary is the fact that Dab1^*Flox*/*Flox*^ mice that exhibit a severe CA1 dispersion in the absence of TMX have a seizure latency which is significantly longer compared to Dab1^*Flox*/*Flox*^ mice treated with TMX. This is particularly remarkable considering the dendritic disorganization found among the ectopically located CA1 pyramidal neurons (Figures 4D–F).

Recent work examining the onset of spontaneous seizure in the pilocarpine model of mTLE in the rat indicates that the firing rate of CA1 increases preictally and may indicate a focal ictal onset zone (Fujita et al., 2014). Despite the dramatic dispersion of CA1, the lack of hyperexcitability leads us to conclude that aberrant migration of CA1 pyramidal cells is not sufficient to drive spontaneous seizures. This further supports the role of the DG in seizure susceptibility and underscores the implications for aberrantly integrated adult-born neurons (Krook-Magnuson et al., 2015).

### Reelin signaling modulates adult neurogenesis

Previous reports have demonstrated a role for reelin signaling in regulating embryonic (Won et al., 2006; Lakoma et al., 2011) and postnatal neurogenesis (Pujadas et al., 2010). We observed a significant decrease in DCX-expressing immature neurons in the adult DG of Dab1-deficient mice, which is novel in this context because the deletion of Dab1 targets NSCs that primarily contribute to ongoing adult neurogenesis. Interestingly, we found no difference in the number of NeuN+/YFP+ cells (data not shown), suggesting that Dab1-deficient NSCs differentiate into neurons at the same rate or higher than intact NSCs. One possible interpretation of these findings is that the percentage of neurons surviving after differentiation increases to compensate for the overall decrease in levels of cell proliferation (Tanapat et al., 2001). Another explanation, not necessary mutually exclusive, is that deletion of Dab1 causes newly-generated neurons to mature faster, resulting in a greater proportion of NeuN+/YFP+ neurons. Prolonged seizures have been shown to have this effect in a mouse model of mTLE (Overstreet-Wadiche et al., 2006), and together with our findings these data further implicate a role for the loss of reelin in structural DG abnormalities associated with mTLE. Future work should be directed at determining how Dab1 deletion influences the survival of newly generated neurons.

Recent work suggests that mice with reduced adult neurogenesis (by x-irradiation or pharmacogenetic deletion) exhibit a potentiated response to chemoconvulsants (Iyengar et al., 2015). Moreover, the pharmacogenetic suppression of adult neurogenesis leads to a potentially compensatory plasticity within the DG involving disinhibition of pre-existing DGCs (Singer et al., 2011). These observations are consistent with our findings that Dab1-deficent mice have dramatically fewer immature neurons and a reduced latency to pilocarpine-induced SE. Our results therefore highlight the importance of adult neurogenesis in maintaining network stability and suggest that this circuit is a potential target for anti-epileptogenic interventions.

## Author contributions

MK and JP all contributed to the design and interpretation of the results. MK and QM collected, processed and quantified data included in this manuscript. MK and JP drafted and revised the contents of this manuscript; MK, QM, and JP approved the final version and its conclusions. MK, QM, and JP agreed to be accountable for the contents of this report.

### Conflict of interest statement

The authors declare that the research was conducted in the absence of any commercial or financial relationships that could be construed as a potential conflict of interest.

## Supplementary material

Supplementary Figure1 **Flowchart of procedures and experimental time points**. Animals were treated with TMX, 2 months later they were fitted for EEG and monitored for 10 days. At the end of the recording period, we challenged the seizure sensitivity with pilocarpine. Once in SE, mice were monitored for 90 min, at which time they were euthanized.
