# Supplementary figures and images for: Conditional Disabled-1 Deletion in Mice Alters Hippocampal Neurogenesis and Reduces Seizure Threshold
Source: Front Neurosci. 2016 Feb 25;10:63. doi: 10.3389/fnins.2016.00063 (PMC4766299; doi:10.3389/fnins.2016.00063)

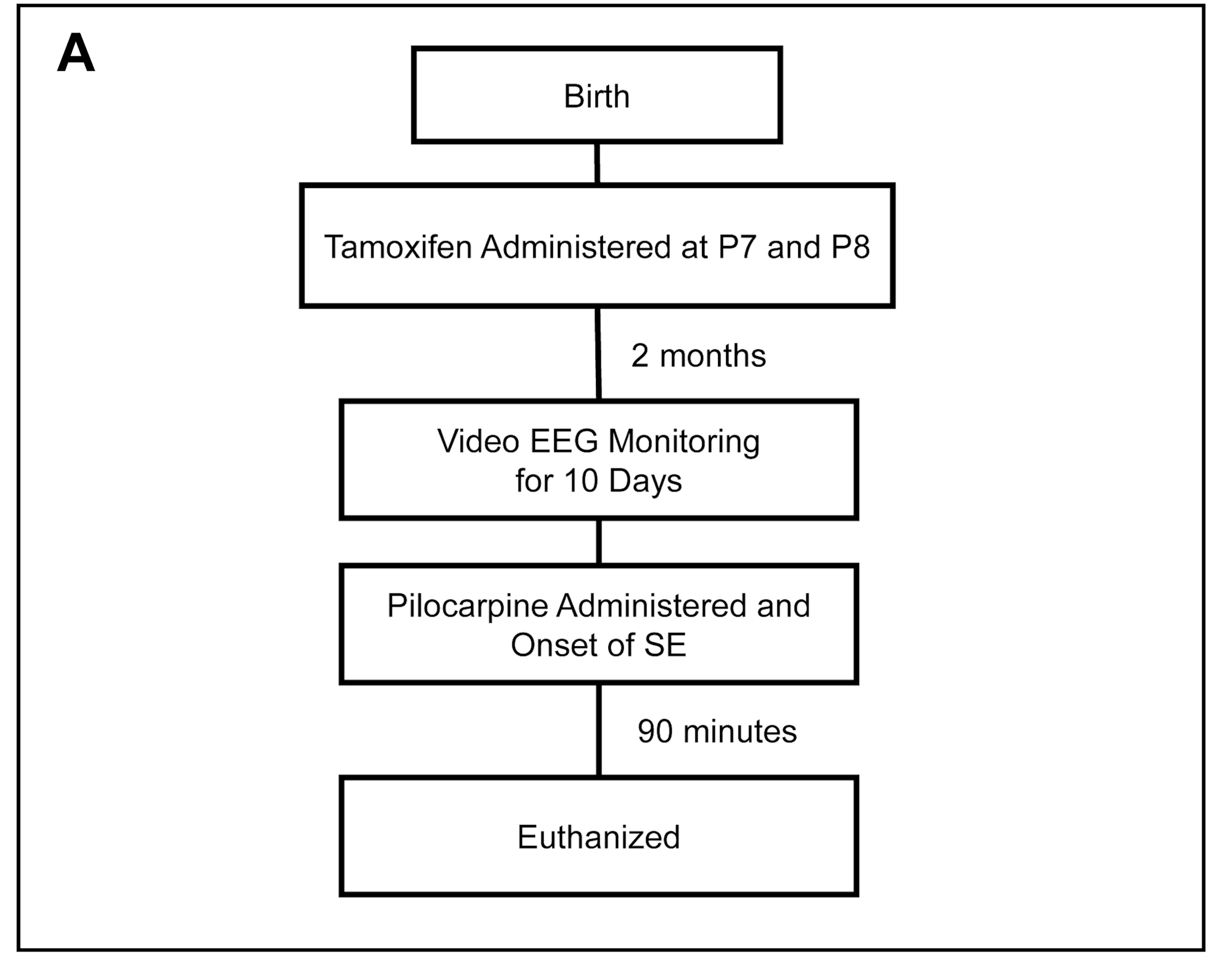

Supplement: Supplementary Figure1 — Flowchart of procedures and experimental time points. Animals were treated with TMX, 2 months later they were fitted for EEG and monitored for 10 days. At the end of the recording period, we challenged the seizure sensitivity with pilocarpine. Once in SE, mice were monitored for 90 min, at which time they were euthanized. [file Image1.TIF]
